# Characterization of endemic *Shigella boydii *strains isolated in Iran by serotyping, antimicrobial resistance, plasmid profile, ribotyping and pulsed-field gel electrophoresis

**DOI:** 10.1186/1756-0500-1-74

**Published:** 2008-08-29

**Authors:** Reza Ranjbar, Caterina Mammina, Mohammad R Pourshafie, Mohammad M Soltan-Dallal

**Affiliations:** 1 Molecular Biology Research Center, Baqiyatallah University of Medical Sciences, Tehran, Iran; 2 Department of Sciences for Health Promotion "G. D'Alessandro", University of Palermo, Via del Vespro 133, I-90127 Palermo, Italy; 3 Department of Microbiology, Pasteur Institute of Iran, Tehran, Iran; 4 Department of Pathobiology, School of Public Health and Institute of Public Health Research, Tehran University of Medical Science, Tehran, Iran

## Abstract

**Background:**

Shigellosis is one of the major causes of morbidity in children with diarrhea in Iran. The present study was undertaken to characterize apparently sporadic *Shigella boydii *strains isolated from pediatric patients in Iran.

**Findings:**

Ten *S. boydii *strains isolated from pediatric cases of gastroenteritis and acute diarrhea in Tehran between December 2002 and November 2003 were submitted to serotyping, antimicrobial susceptibility testing, plasmid profile analysis, ribotyping and pulsed field gel electrophoresis (PFGE). Seven isolates were attributed to serotype 2, whereas the remaining three belonged to serotypes 14, 18, 19, respectively. Six drug resistance phenotypes (R1 to R6) were defined with R4 – streptomycin (STR), ampicillin (AMP), sulfamethoxazole-trimethoprim (SXT) – being the most prevalent. Plasmid analysis resulted in seven different plasmid profiles with one to five DNA bands. All strains, but one, shared the same ribotype, but PFGE differentiated them in four groups.

**Conclusion:**

Based upon ribotyping and PFGE results, endemic circulation of *S. boydii *in Tehran, Iran, could be attributed to a few clones. Resistance pattern and plasmid profile analysis proved to be very effective in discriminating apparently unrelated strains of *S. boydii*.

## Findings

Shigellosis is one of the major causes of morbidity and mortality in children with diarrhea in developing countries. Worldwide, the disease causes around 1,100,000 deaths per year, and two-thirds of the patients are children under 5 years of age. Shigellosis is caused by four serogroups of *Shigella *including serogroup A (*S. dysenteriae*), serogroup B (*S. flexneri*), serogroup C (*S. boydii*), and serogroup D (*S. sonnei*). Among *Shigella *serogroups, *S. flexneri *and *S. sonnei *are most prevalent in the developing and industrialized countries, respectively. *S. dysenteriae *is detected mostly in South Asia and sub-Saharian Africa [1]. *S. boydii *has been less frequently reported worldwide compared to other *Shigella *serogroups [2,3]. It is relatively rare in developed countries and is typically associated with individuals who have travelled to endemic areas. Isolation rate of this species is less than 1–2% of the total *Shigella *isolates, except in the Indian subcontinent [4].

Shigellosis is one of the major causes of morbidity in children with diarrhea in Iran [5-9], but reports about prevalence of *Shigella *serogroups and their phenotypic and genetic epidemiological features are limited. Furthermore, specific studies to investigate the relatedness between the *S. boydii *strains isolated from clinical cases in Iran have never been undertaken.

Several typing methods such as serotyping, drug resistance pattern, plasmid analysis, ribotyping, and pulsed field gel electrophoresis (PFGE) have been frequently used for subtyping of *Shigella *in epidemiological investigations [3,10-14].

The present study was undertaken to characterize *S. boydii *strains isolated from pediatric cases of gastroenteritis and acute diarrhea in Tehran, Iran, between December 2002 and November 2003 by serotyping, antimicrobial susceptibility testing, plasmid profile analysis, ribotyping and PFGE. To the best of our knowledge this is the first study of its kind to be carried out in Iran.

## Methods

### Bacterial strains

Fecal specimens and rectal swabs were inoculated into Cary-Blair transport medium and processed within 2–4 h. Specimens were cultured on *Shigella-Salmonella *(SS), Hektoen-Enteric (HE), Xylose Lysine Deoxycholate (XLD) and MacConkey (MC) agars (Difco, Detroit, MI, USA) and incubated at 37°C for 24 h. Suspected colonies were picked from the culture plates and subjected to further analysis by biochemical tests for the identification of possible *Shigella *colonies. *Shigella *spp. were preliminarily identified by Gram stain, colony morphology, lactose fermentation, motility, as well as by API20E [15].

For serotyping, strains of *Shigella *were subcultured on trypticase soy agar (Difco, Detroit, MI, USA) and tested for agglutination on glass slides. Strains were serogrouped by using commercially-available polyclonal antisera from MAST Group LTD (Mast House, Derby Road, Bootle, Merseyside, L201EA). The serotypes of *S. boydii *were determined with commercially available monovalent antisera (Staten Serum Institut, Copenhagen, Denmark).

### Antimicrobial susceptibility testing

Antimicrobial susceptibility test was performed by the disk diffusion method according to the guidelines of the Clinical and Laboratory Standards Institute (formerly National Committee for Clinical Laboratory Standards [NCCLS], 2000) [16]. The following antimicrobial agents were tested; ampicillin, AMP (10 μg); amoxicillin-clavulanic acid, AMX (30 μg); cephalothin, CF (30 μg); cefixime, CFM (5 μg); ceftazidime, CAZ (30 μg); ceftizoxime, CT (30 μg); ceftriaxone, CRO (30 μg); amikacin, AN (30 μg); gentamicin, GM (10 μg); kanamycin, K (30 μg); streptomycin, STR (10 μg); chloramphenicol, C (30 μg); sulfamethoxazole-trimethoprim, SXT (23.75/1.25/μg); tetracycline, TET (30 μg); ciprofloxacin, CP (5 μg); nalidixic acid, NA (30 μg). *Escherichia coli *ATCC 25922 was used as a quality control strain.

### Plasmid profiling

The High Pure plasmid isolation kit (Roche, Mannheim, Germany) was used to isolate the bacterial plasmids in accordance with the manufacturer's recommendations. This extraction procedure is suitable for detection of small plasmids (less than 20 kbp molecular size) only. However, since large virulence plasmids carried by all four *Shigella *serogroups are associated to virulence properties and do not contribute to the discriminative power [11,17,18], the method was considered to be reliable for subtyping purposes.

Plasmid DNA was then separated on a 0.8% agarose gel in Tris-borate-EDTA (TBE) buffer, pH 8.2, by horizontal electrophoresis. The strains were grouped depending on their banding pattern. Lambda DNA cleaved by *Eco*R1 and *Hin*dIII (Promega, Madison, WI, US) was used as electrophoresis marker.

### Ribotyping

Ribotyping was performed according to previous reports [19]. Bacterial DNA was digested with *Pvu*II restriction enzyme under conditions recommended by the manufacturer (Roche Diagnostics, Mannheim, Germany). Digested DNA fragments were resolved on a 0.8% agarose gel in TBE buffer and then transferred onto nylon membrane by the alkali-blotting procedure on a vacuum blotter. Hybridization was then performed with a digoxigenin-11-dUTP (DIG) labeled oligonucleotide probe mixture [20]. The membranes were then visualized by addition of alkaline phosphate-conjugated anti-digoxigenin antibody (Roche Diagnostic GmbH, Mannheim, Germany) and nitroblue tetrazolium and 5-bromo-4-chloro-3-indolyl phosphate as the substrate.

### PFGE analysis

The *S. boydii *isolates were analyzed by PFGE after DNA digestion with the restriction endonuclease *Xba*I (Promega, Madison, WI, USA) using the PulseNet protocol [21]. DNA of *Salmonella *serotype Braenderup strain H9812 was digested with *Xba*I and used as molecular weight standard. Strain H9812 was kindly provided by the National Reference Centre for Enteric Pathogens at the Istituto Superiore di Sanità, Rome, Italy. The electrophoretic profiles were visually compared and interpreted according to the criteria of Tenover *et al*. [22].

## Results

During the one-year period from December 2002 and November 2003, a total of 302 *Shigella *strains had been isolated from enteritis cases in children at the Children Medial Center and Mofid Children Hospital and three additional large hospitals including Baqiyatallah, Millad and Firozabadi in Tehran.

Ten isolates of *S. boydii *were available for the study. No epidemiological relationship had been identified within the strains by conventional investigation.

Seven strains belonged to serotype 2, whereas serotypes 14, 18, and 19 were each represented by one strain.

All isolates were resistant to sulfamethoxazole-trimethoprim and all, but one, to streptomycin. Additional resistances to nalidixic acid or ampicillin were exhibited by four isolates. The majority of the isolates were susceptible to amoxicillin-clavulanic acid, chloramphenicol and tetracycline. All isolates were fully susceptible to kanamycin, gentamicin, cephalothin, ceftriaxone, ceftizoxime, ceftazidime, cefotaxime and ciprofloxacin. Table 1 summarizes the antimicrobial resistance patterns.

**Table 1:** Characteristics of the *S. boydii *isolates included in the study

**Strain No**.	**Serotype**	Resistance pattern*	**Plasmid pattern**	**Ribotype**	**PFGE pattern**	**Date of isolation**
1	2	STR (R1)	P6	I	A	Dec 2002
2	19	STR, SXT, NDA (R2)	P1	I	B	Feb 2002
3	2	STR, SXT, NDA, TET (R3)	P2	I	A	May 2003
4	2	STR, SXT, AMP, (R4)	P3	I	A	Aug 2003
5	2	STR, SXT, NDA, TET (R3)	P4	I	A	Sep 2003
6	14	STR, SXT, NDA, TET, AMX, C, AMP (R5)	P5	II	C	Sep 2003
7	2	STR, SXT, AMP (R4)	P6	I	A	Oct 2003
8	18	STR, SXT (R6)	P7	I	D	Oct 2003
9	2	STR, SXT (R6)	P6	I	A	Nov 2003
10	2	STR, SXT, AMP (R4)	P6	I	A	Nov 2003

*AMP, ampicillin; AMX, amoxicillin-clavulanic acid; C, chloramphenicol; NDA, nalidixic acid; STR, streptomycin; TET, tetracycline; SXT, sulfamethoxazole-trimethoprim.

**Figure 1:**
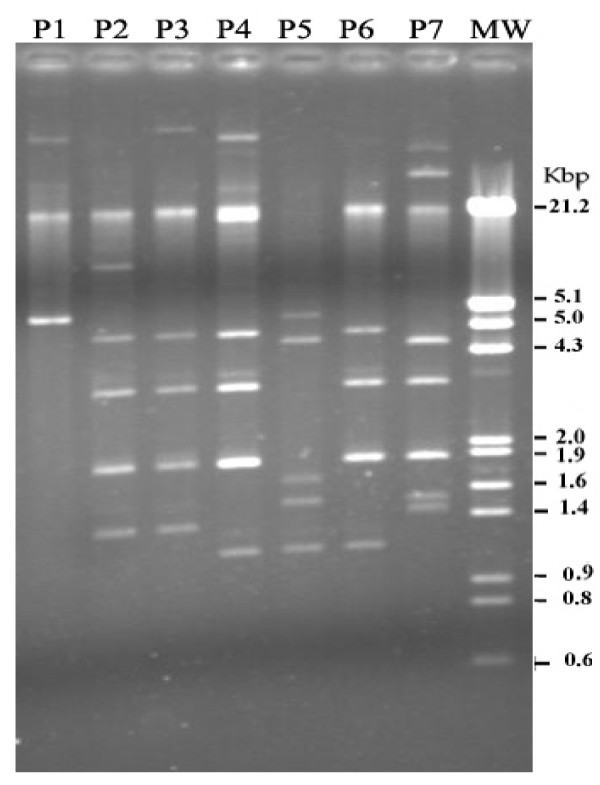
Plasmid profiles of *S. boydii *isolates. P1–P7 are the representative plasmid profiles of the clinical samples. Lane M is molecular size marker.

Plasmid analysis of *S. boydii *resulted in seven different plasmid profiles (P1 to P7) with one to five DNA bands with a molecular weight lower than approximately 20 kb (Fig. 1). The most prevalent plasmid profile was P6 (40%). Other plasmid profiles were unique (Table 1).

Drug resistance patterns combined with plasmid profiles stratified the ten *S. boydii *isolates into nine groups.

**Figure 2:**
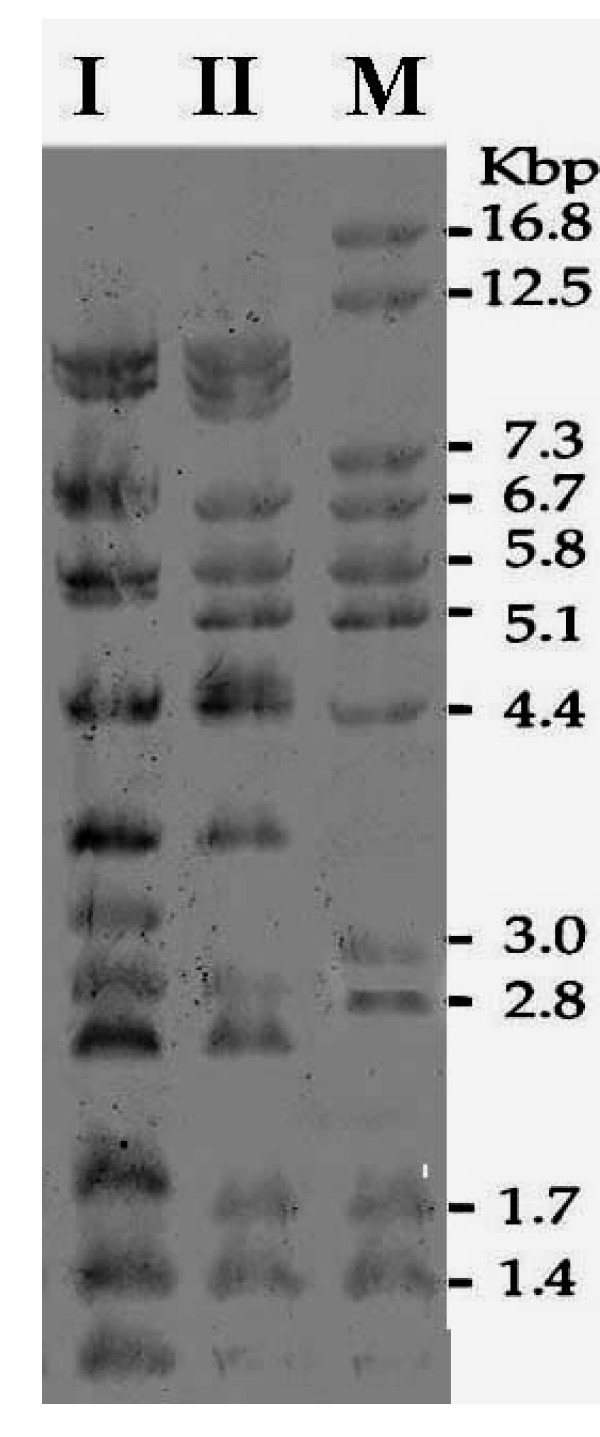
Ribotyping of *S. boydii *(*Pvu*II digestion and Southern blotting). Lanes I and II are the representative ribotypes of the clinical strains. *Citrobacter koseri *DNA was digested by *Mlu*I and served as a molecular size marker (M).

Ribotyping produced 14 fragments when DNA from all *S. boydii *isolates was digested with *Pvu*II. Two ribotype patterns were observed, of which ribotype I include nine isolates and ribotype II one only isolate (Fig. 2).

**Figure 3:**
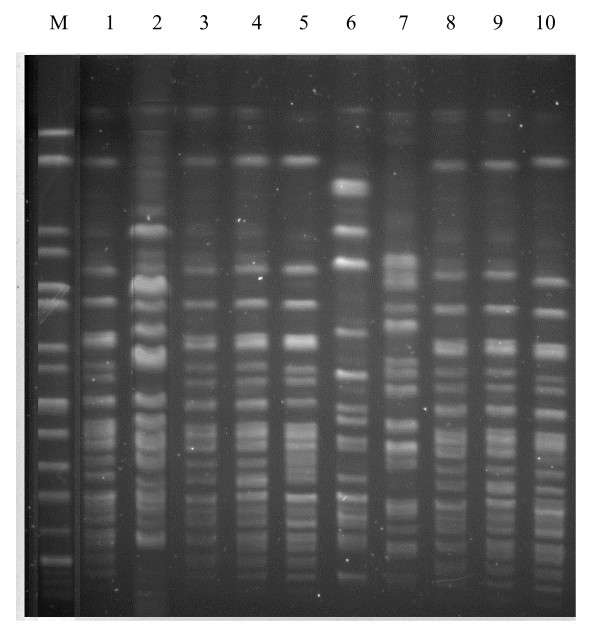
PFGE of *Xba*I-digested genomic DNAs from *S. boydii *isolates. Lane M is *Xba*I-digested genomic DNA from *Salmonella *serotype Braenderup strain H9812 which served as a molecular size marker. Lanes: 1, 3–5, 7–10, strains 1, 3, 4, 5, 7, 9 and 10, PFGE pattern A; 2, strain 2, PFGE pattern B; 6, strain 6, PFGE pattern C; 7, strain 8, PFGE pattern D.

Four pulsotypes, named A to D, were identified by *Xba*I-PFGE among the isolates under study (Fig. 3). Type A included seven isolates, whereas types B, C and D one isolate each, respectively.

The results of the different subtyping methods are illustrated by the Table 1. Combining results of the phenotypic and genetic subtyping methods, nine different complete patterns were identified.

## Discussion

Of 302 *Shigella *isolates recovered from Iranian pediatric cases in the period December 2002 to November 2003, 10 (3.3%) strains were identified as *S. boydii *and analyzed in this study by different phenotypic and molecular subtyping methods. The prevalence rate of *S. boydii *is comparable with a recent study in Iran in which 3.1% of *Shigella *strains belonged to this serogroup [9].

Antimicrobial drug resistance pattern analysis has been widely applied in epidemiologic studies of *Shigella *through the years [23]. Using this method, six resistant phenotypes were recognized with resistance to streptomycin, ampicillin and sulfamethoxazole-trimethoprim being the most frequent pattern. Our results suggest that this method may represent an undemanding, but highly discriminative approach for differentiation of *S. boydii*. Moreover, it is the least expensive and most widely available subtyping tool and could be considered as a preliminary screening approach in assessing strain relatedness. Nevertheless, significance of drug resistance profiles in long-term epidemiologic studies is limited by two concurrent factors: the strong selective pressure caused by antibacterial drug abuse/misuse in hospital and, increasingly, in community settings and the phenotype instability [24,25]. Indeed, all *S. boydii *strains were resistant to trimethoprim-sulfamethoxazole and all, but one, to streptomycin, antibacterial drugs that are both commonly used in Iran as an empiric therapy in the treatment of shigellosis and the other bacterial enteric diseases. Furthermore, the changes in resistance phenotypes that have been observed in *Shigella *have recently been supposedly attributed to the transposition activity of insertion sequences distributed at high frequency through the genome with a consequent disruption of antimicrobial resistance sequences [25].

Plasmid profile analysis has been extensively used on *Shigella *strains. This method is cheap and quick, requiring one hour approximately of hands-on time, and 24 hours to be completed. *Shigella *species may harbor plasmids ranging from 2 to as many as 10 different populations [26,27]. Some previous studies used plasmid profiles to characterize isolates of *S. boydii *[3,18,9]. The sizes of the plasmids among all *S. boydii *isolates ranged from 1 to 21.2 kb that is comparable with the results previously reported from USA [18] and Iran [9]. In our study, plasmid profile analysis was able to differentiate seven patterns among 10 isolates: P6 only was shared by four strains, while the remaining plasmid profiles were unique. Litwin *et al*. [18] also found six plasmid patterns among 12 strains of *S. boydii *isolated from Pima County, Arizona between April 1986 and May 1987. Those patterns contained one to five plasmids which ranged in size from 1.4 to >20 kb. In other studies, however, plasmid profile analysis showed an identical plasmid pattern in 23 of 25 strains studied [3]. In a recent study carried out in Shiraz, Iran, Farshad and her colleagues identified three genotypes among three clinical isolates of *S. boydii *on the basis of their plasmid profiles [9].

Ribotyping was applied based upon previous findings proving that rDNA restriction pattern analysis may be a valuable tool in epidemiological research on *S. boydii *[3,10]. In our study, however, all *S. boydii *strains were categorized into two patterns only. Moreover, all but one isolates, belonged to ribotype I. Hence, in our setting ribotyping did not prove to be useful, despite the encouraging results obtained on *Shigella *during previous investigations [12,28]. However, our finding is similar to recent reports from other developing countries, such as Bangladesh [29].

PFGE also, that has consistently proved to be a highly discriminative subtyping method in epidemiological investigation of many bacterial pathogens [30], divided our strains into four groups closely associated to serotype. The association of the serotype 2/PFGE A pattern subtype with different plasmid profiles could suggest the circulation during the period under study of a major endemic *S. boydii *clone, including strains that were going through some differentiation steps probably by acquisition or loss of extra-chromosomal DNA.

Though our results have inherent limits due to the lowest incidence of *S. boydii *infections in Iran and the consequent small number of available strains, subtyping relying upon more temporally and spatially stable molecular markers, such as PFGE and ribotyping, suggests that endemic circulation of this enteric pathogen should be attributed to a few bacterial clones. Simultaneously, less stable markers, such as drug resistance pattern and plasmid profile analysis, were very effective in finely discriminating apparently unrelated strains of *S. boydii*.

## Conclusion

Low endemic circulation of *S. boydii *in Tehran, Iran, may be attributed to a few clones diverging towards heterogeneous drug resistance phenotypes and plasmid profiles.

The different methods could provide more or less sensitive interpreting keys suitable for long term – ribotyping, PFGE – or short term – drug resistance and plasmid pattern – epidemiological studies.

To our best knowledge, this is the first report on the characterization by different methods of *S. boydii *strains isolated in Iran. The results obtained from present study could be helpful for a future epidemiological surveillance of *Shigella *in this country.

## Competing interests

The authors declare that they have no competing interests.

## Authors' contributions

RR conceived the study, carried out bacterial isolation, drug resistance analysis, plasmid profiling, ribotyping and drafted the manuscript. CM participated in study design and coordination, carried out serotyping, helped to PFGE analysis and draft of the manuscript. MRP and MMSD helped to study design and draft of the manuscript. All authors read and approved the final manuscript.
